# Epigenetic Clocks of Biological Aging and Risk of Incident Mild Cognitive Impairment and Dementia: The Women's Health Initiative Memory Study

**DOI:** 10.1111/acel.70424

**Published:** 2026-02-21

**Authors:** Steve Nguyen, Ake T. Lu, Steve Horvath, Mark A. Espeland, Stephen R. Rapp, Adam X. Maihofer, Caroline M. Nievergelt, Andrea Z. LaCroix, Linda K. McEvoy, Susan M. Resnick, Kenneth Beckman, Aladdin H. Shadyab

**Affiliations:** ^1^ Herbert Wertheim School of Public Health and Human Longevity Science University of California San Diego La Jolla California USA; ^2^ Altos Labs San Diego California USA; ^3^ Division of Gerontology and Geriatric Medicine, School of Medicine Wake Forest University Winston‐Salem North Carolina USA; ^4^ Department of Psychiatry and Behavioral Medicine Wake Forest School of Medicine Winston‐Salem North Carolina USA; ^5^ Department of Psychiatry University of California San Diego La Jolla California USA; ^6^ Research Service, Veterans Affairs San Diego Healthcare System San Diego California USA; ^7^ Kaiser Permanente Washington Health Research Institute Seattle Washington USA; ^8^ Laboratory of Behavioral Neuroscience National Institute on Aging, National Institutes of Health Baltimore Maryland USA; ^9^ Genomics Center University of Minnesota Minneapolis Minnesota USA; ^10^ Division of Geriatrics, Gerontology, and Palliative Care, Department of Medicine University of California San Diego San Diego California USA

**Keywords:** aging, biomarkers, cohort studies, dementia, epigenomics

## Abstract

Aging is the strongest risk factor for dementia; however, few studies have examined the association of biological aging with incident dementia. We analyzed 6069 cognitively unimpaired women (mean age = 70.0 ± 3.8 years) in the Women's Health Initiative Memory Study to examine the association of accelerated biological aging, measured with second and third‐generation epigenetic clocks (AgeAccelPheno and AgeAccelGrim2, and DunedinPACE, respectively) with incident mild cognitive impairment (MCI) and probable dementia. Multivariable Cox proportional hazards models adjusted for age, education, race, ethnicity, smoking, hormone therapy regimen, physical activity, body mass index, and estimated white blood cell counts. For comparison, we also examined first‐generation epigenetic clocks (AgeAccelHorvath; AgeAccelHannum). We evaluated effect modification by age, race/ethnicity, hormone therapy regimen, menopause type (natural vs. surgical), and *APOE ε4* carriage. There were 1307 incident MCI or probable dementia events over a median follow‐up of 9.3 (25th percentile = 6.1, 75th percentile = 16.1) years. The adjusted HRs (95% CI; *p*‐value) for incident MCI/probable dementia per one‐standard deviation increment were 1.07 (1.01–1.15; *p* = 0.03) for DunedinPACE, 1.11 (1.02–1.20; *p* = 0.01) for AgeAccelGrim2, and 1.01 (0.95–1.07; *p* = 0.74) for AgeAccelPheno. Only AgeAccelGrim2 remained significant under the Bonferroni‐corrected threshold for significance (*p* < 0.02). Other epigenetic clocks were not associated with incident MCI/probable dementia. There was no effect modification in most subgroup analyses (*p*‐interaction ≥ 0.05). In this cohort study of older women, accelerated biological aging measured by AgeAccelGrim2 was associated with higher risk of incident MCI/probable dementia. These findings provide evidence linking epigenetic biomarkers of biological aging with MCI and dementia development, independent of chronological age.

## Graphical abstract

In this prospective cohort study of 6069 older women, accelerated biological aging captured by the epigenetic age biomarker AgeAccelGrim2 was associated with a higher risk of incident MCI/probable dementia. These findings provide evidence linking epigenetic biomarkers of biological aging with MCI and dementia development.

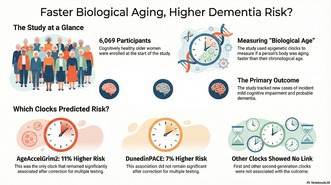

## 1 Introduction

Aging is the strongest risk factor for Alzheimer's disease (AD) and related dementias (Alzheimer's Association 2025, n.d.). Over two‐thirds of AD cases are women. The estimated lifetime risk of AD among women aged 65 or older is 21.1% (nearly twice that of men at 11.6%), in part due to women living longer than men on average (Chene et al. 2015). Women therefore represent a key population for understanding the heterogeneity of aging and dementia risk.

Epigenetic clocks are DNA methylation (DNAm)‐based measures of biological aging that could inform on the heterogeneity of aging and dementia risk. Several studies have shown that epigenetic age acceleration, indicating faster biological aging relative to chronological age, is associated with higher risk of age‐related outcomes including cardiovascular disease and mortality (Chen et al. 2016; Forrester et al. 2024). First‐generation clocks including Horvath and Hannum DNAm age were trained on chronological age (Hannum et al. 2013; Horvath 2013). Second‐generation clocks including DNAm PhenoAge, DNAm GrimAge, and GrimAge2 were developed to predict phenotypic age and mortality, respectively (Levine et al. 2018; Lu et al. 2019, 2022, 2). Third‐generation clocks, such as DunedinPACE, were developed to estimate the pace of biological aging using longitudinal data on biomarkers of organ system functioning (Belsky et al. 2022).

Yet, few large studies have examined the association of accelerated biological aging assessed with next‐generation clocks, such as AgeAccelGrim2 and DunedinPACE, with incident mild cognitive impairment (MCI) and dementia. Prior studies had limited sample sizes, few MCI or dementia cases, less than 2 decades of follow‐up, or examined few epigenetic clocks (Shadyab et al. 2022; Sugden et al. 2022). Therefore, larger and more rigorous studies with longer follow‐up periods are warranted.

We previously published preliminary findings suggesting potential associations of epigenetic age acceleration with cognitive outcomes among a subset of 578 women from the Women's Health Initiative (WHI) Memory Study (WHIMS); however, we examined only four clocks and did not examine third‐generation clocks (Shadyab et al. 2022). In this larger, well‐powered study, we examined the associations of first‐, second‐, and third‐generation epigenetic clocks with incident MCI and dementia in 6069 WHIMS women with up to 25 years of follow‐up, performing the largest study on epigenetic clocks and incident MCI and dementia to date.

## 2 Methods

### 2.1 Study Population

The data supporting the present study's findings are available on reasonable request from the WHI Program per publications and presentations policies (https://www.whi.org/doc/PP‐policy.pdf).

WHIMS, an ancillary study of the WHI Hormone Trials, was designed to investigate the effects of hormone therapy (conjugated equine estrogens (CEE) alone vs. placebo or CEE plus medroxyprogesterone [E + P] acetate vs. placebo) on cognitive outcomes among 7479 women aged 65–80 years recruited from 39 clinical sites across the United States who were cognitively unimpaired at randomization (1996–1999) (Shumaker et al. 1998). WHIMS administered annual in‐person cognitive assessments during follow‐up through 2007 for MCI and dementia (Shumaker et al. 1998). In 2008, WHIMS transitioned to annual telephone‐based cognitive assessments in the WHIMS‐Epidemiology of Cognitive Health Outcomes (WHIMS‐ECHO) study, with follow‐up for MCI and probable dementia through 2021 (Espeland et al. 2017).

In the present study, we excluded 240 participants with only 1 WHIMS cognitive assessment, 519 who were ineligible for dbGaP, and 304 without available baseline DNA or buffy coat, resulting in 6416 WHIMS women with DNAm data. After DNAm quality control (see Methods S1), 347 women were excluded, resulting in 6069 women who were free of MCI and probable dementia at baseline. The WHI Clinical Coordinating Center at Fred Hutchinson Cancer Center (Seattle, WA) approved all study protocols. This study was approved by the Institutional Review Board of University of California, San Diego. All women provided informed written consent. This study conforms to the Strengthening the Reporting of Observational Studies in Epidemiology (STROBE) guidelines.

### 2.2 MCI and Probable Dementia Outcomes

Our primary outcome was the combined endpoint of MCI or probable dementia, whichever came first. We also examined MCI and probable dementia separately as secondary outcomes. MCI and probable dementia were not mutually exclusive as participants could have developed MCI and later probable dementia, contributing an event to each respective secondary analysis. Details on MCI and probable dementia adjudication in WHIMS are published elsewhere and briefly described below: (Shumaker et al. 1998).

Participants completed the Modified Mini Mental State Examination (3MSE) in‐person annually through 2007. Those scoring below age and education adjusted cutoffs completed a modified Consortium to Establish a Registry for Alzheimer's Disease battery of neuropsychological and standardized tests (Shumaker et al. 1998). A physician at each study site with expertise in diagnosing dementia then classified women with MCI, probable dementia, or no cognitive impairment using Petersen's criteria for MCI and Diagnostic and Statistical Manual of Mental Disorders, Fourth Edition criteria for probable dementia (American Psychiatric Association 1994; Petersen et al. 2001). All participant data were sent to the WHIMS Clinical Coordinating Center for central adjudication by an expert panel consisting of a neurologist, geriatric psychiatrist, and geropsychologist (Shumaker et al. 1998).

WHIMS‐ECHO used a battery of validated neurocognitive tests including the Telephone Interview for Cognitive Status‐modified (TICS‐m), which measures global cognitive function similar to the 3MSE (Espeland et al. 2017; Rapp et al. 2012). For women who scored below age and education adjusted cutoffs on the TICS‐m, an interview with the participant's pre‐identified proxy was conducted to obtain required information about her functional status. Otherwise, the protocol for diagnosing and centrally adjudicating MCI, probable dementia, or no cognitive impairment was the same as that of WHIMS.

The WHIMS‐Supplemental Case Ascertainment Protocol (SCAP) was implemented to identify MCI and probable dementia cases in deceased or proxy‐dependent participants (Gaussoin et al. 2012, 2019). The Dementia Questionnaire was administered to the participant‐identified proxy respondent (Gaussoin et al. 2012, 2019). SCAP and prior WHIMS data were submitted to the central adjudication panel for final ascertainment of MCI/probable dementia, similar to WHIMS (Gaussoin et al. 2012, 2019).

### 2.3 Epigenetic Clocks

Details on DNAm measurement are provided in the Supplemental Methods. For our main analysis, we focused on 2 extensively studied second‐generation clocks, AgeAccelPheno and AgeAccelGrim2, and a third‐generation clock, DunedinPACE. We focused on these clocks because they have been shown to have stronger associations with age‐related phenotypes compared with first‐generation clocks (Belsky et al. 2022; Conole et al. 2025; Levine et al. 2018; Lu et al. 2022; Sugden et al. 2022). For comparison, we also examined 2 first‐generation clocks, AgeAccelHorvath and AgeAccelHannum (Hannum et al. 2013; Horvath 2013). In secondary analyses, we examined intrinsic and extrinsic epigenetic age acceleration (IEAA and EEAA, respectively). We also examined principal component (PC)‐based versions of the first‐ and second‐generation clocks (PCHorvath, PCHannum, PCPhenoAge, and PCGrimAge) because published studies have shown that PC‐based clocks showed superior reliability with intraclass correlation coefficients of > 0.97 for age acceleration measures compared with the original clocks (Higgins‐Chen et al. 2022). We also examined the 10 DNAm‐based AgeAccelGrim2 components individually (Lu et al. 2022). Details on calculation of the epigenetic clocks are provided in the Supplemental Methods.

For the epigenetic clocks, higher values indicate accelerated biological aging relative to chronological age. For DunedinPACE, values > 1 indicate a faster pace of aging (e.g., a value of 1.10 indicates a pace of aging 10% faster than the average), while values < 1 indicate a slower pace of aging (e.g., a value of 0.90 indicates a pace of aging 10% slower than the average) (Belsky et al. 2022).

### 2.4 Covariates

Baseline questionnaires assessed age, race, ethnicity, education, smoking status, and total energy expenditure from recreational physical activity (metabolic equivalent hours/week). Height was measured with a stadiometer and weight with a balance beam scale to calculate body mass index (BMI; kg/m^2^). Menopause type was classified as natural or surgical (Shadyab et al. 2017). We defined *APOE ε4* carriage as presence of at least 1 *ε4* allele, which was determined among White women with available genome‐wide genotyping data based on 2 single nucleotide polymorphisms: rs429358 and rs7412. Imputation was performed using the 1000 Genomes Project reference panel, and MaCH algorithms were implemented in Minimac (Howie et al. 2012). White blood cell (WBC) counts were estimated using IDOL (Salas et al. 2022).

### 2.5 Statistical Analysis

We calculated means and standard deviations for continuous variables or counts and proportions for categorical variables. We compared baseline characteristics across quartiles of the three clocks of primary interest in our study, including AgeAccelGrim2, DunedinPACE, and AgeAccelPheno separately, using *F*‐tests for continuous variables or chi‐squared tests for categorical variables. We also compared characteristics between participants included in the analytic sample (*n* = 6069) and WHIMS women who were excluded (*n* = 1410). We calculated Pearson correlations between all epigenetic clocks. We estimated Kaplan–Meier curves across tertiles of each of the 5 main clocks we examined and capped follow‐up at the 90th percentile due to smaller risk sets from censoring and unstable survival estimates. Differences in survival across tertiles were assessed using log‐rank tests.

Multivariable Cox proportional hazards regression models estimated hazard ratios (HR) and 95% confidence intervals (CI) for the associations of one‐standard deviation (SD) increments in epigenetic clocks to facilitate comparisons of study results across clocks with different units of measurement, with the main endpoint of combined MCI/probable dementia and secondary outcomes of MCI and probable dementia separately. We calculated *p*‐values for epigenetic clock associations using the Wald test for the regression coefficient in the models. We calculated follow‐up time as days from baseline to the date of the cognitive assessment that triggered the first diagnosis of MCI or probable dementia (whichever came first for combined MCI/probable dementia), or the date of the last cognitive assessment, similar to prior WHIMS studies (Shumaker et al. 2003, 2004). For participants in WHIMS‐SCAP, those with MCI or probable dementia had their follow‐up time set to the midpoint between the date of their last cognitive assessment and Dementia Questionnaire if they were alive, or date of death if deceased (Gaussoin et al. 2012, 2019). For those without MCI or probable dementia from WHIMS‐SCAP, follow‐up time was defined as the later of the date of their last cognitive assessment or Dementia Questionnaire if they were alive, or date of death if deceased (Gaussoin et al. 2012, 2019). Schoenfeld residuals tested the proportional hazards assumption; no violations were observed.

Model 1 adjusted for age. Model 2 additionally included race and ethnicity as separate variables including all categories, as published studies have shown differences in epigenetic clocks, MCI, and dementia by race and ethnicity (Crimmins et al. 2021; Kornblith et al. 2022). Model 3 additionally included potential confounders informed by the published literature: education, hormone therapy trial arm, smoking, BMI, and physical activity. The fully adjusted model (Model 4) additionally included estimated WBC counts. Models with IEAA, IEAA.Hannum, or EEAA did not contain WBC counts since IEAA was derived to be independent of WBC counts, while EEAA incorporates them into its calculation. To account for missing covariate data, we applied multiple imputation by chained equations using the R *mice* package, specifying all study variables with 100 imputations and 5 iterations.

In sensitivity analyses, we repeated the main models excluding women who had MCI/probable dementia during the first 2 years of follow‐up to mitigate the potential of reverse causation bias on the primary results. To evaluate the consistency of associations across cohort subgroups, we performed stratified analyses across age, hormone therapy trial arm, menopause type (natural vs. surgical), race/ethnicity (Black, Hispanic or Latina, or White), and *APOE ε4* carriage. We statistically evaluated effect modification using cross‐product interaction terms between epigenetic clocks and stratification variables.

We report nominal *p*‐values for all tests. For completeness, we note that the Bonferroni‐corrected threshold for the 3 main clocks of interest (AgeAccelPheno, AgeAccelGrim2, and DunedinPACE) and the primary outcome of MCI/probable dementia would be 0.0167 (0.05/3). We analyzed the data using R 4.4.1 from September 2024 to September 2025.

## 3 Results

At baseline, women were 70.0 years on average, 0.2% were American Indian or Alaskan Native, 1.7% were Asian, 0.1% were Native Hawaiian or other Pacific Islander, 6.9% were Black, 89% were White, 1.1% reported more than one race, and 0.9% were unknown or not reported race (Table 1). Overall, 2.6% reported Hispanic or Latina ethnicity. Compared with women in the lowest quartile of AgeAccelGrim2, those in the highest quartile were less likely to be White, less likely to have higher educational attainment, more likely to smoke, had higher BMI, and reported less physical activity (Table 1). A generally similar pattern of differences across quartiles was noted for DunedinPACE and AgeAccelPheno (Tables S1 and S2). However, women in the highest quartile of AgeAccelPheno were more likely to be White compared to those in the lowest (Table S2). Compared with women in the analytic sample, women who were excluded were older, were more likely to be Hispanic or Latina, were less likely to have high educational attainment, and were similar across race, smoking, BMI, physical activity, and *APOE ε4* carriage (Table S3).

**TABLE 1:** Baseline sociodemographic, behavior, and health characteristics by quartiles of AgeAccelGrim2 in the Women's Health Initiative Memory Study (*n* = 6069).

Characteristics	Total	AgeAccelGrim2 quartiles				*p*
		Q1 (*n* = 1518): [−14.4, −2.97]	Q2 (*n* = 1517): (−2.97, −0.56]	Q3 (*n* = 1517): (−0.56, 2.30]	Q4 (*n* = 1517): (2.30, 21.7)	
	No. (%)	No. (%)	No. (%)	No. (%)	No. (%)	
Age, mean (SD)	70.0 (3.8)	69.8 (3.7)	70.0 (3.8)	70.5 (3.9)	69.9 (3.9)	< 0.001
Highest education level, *n* (%)						
Less than high school/GED	421 (7)	76 (5)	93 (6.1)	100 (6.6)	152 (10.1)	< 0.001
High school/GED	1306 (21.6)	332 (22)	324 (21.4)	331 (21.9)	319 (21.1)	
Some college	2414 (39.9)	568 (37.6)	593 (39.2)	612 (40.5)	641 (42.5)	
College graduate or higher	1906 (31.5)	536 (35.4)	504 (33.3)	468 (31)	398 (26.4)	
Hormone trial arm						
Estrogen alone intervention	1152 (19)	250 (16.5)	301 (19.8)	304 (20)	297 (19.6)	0.003
Estrogen placebo	1163 (19.2)	253 (16.7)	298 (19.6)	289 (19.1)	323 (21.3)	
Estrogen plus progestin intervention	1854 (30.5)	489 (32.2)	456 (30.1)	456 (30.1)	453 (29.9)	
Estrogen plus progestin placebo	1900 (31.3)	526 (34.7)	462 (30.5)	468 (30.9)	444 (29.3)	
Race, *n* (%)						
American Indian or Alaskan Native	13 (0.2)	4 (0.3)	4 (0.3)	1 (0.1)	4 (0.3)	< 0.001
Asian	106 (1.7)	32 (2.1)	29 (1.9)	24 (1.6)	21 (1.4)	
Native Hawaiian or other Pacific Islander	8 (0.1)	2 (0.1)	2 (0.1)	2 (0.1)	2 (0.1)	
Black	417 (6.9)	54 (3.6)	74 (4.9)	115 (7.6)	174 (11.5)	
White	5403 (89)	1400 (92.2)	1380 (91)	1343 (88.5)	1280 (84.4)	
More than 1 race	68 (1.1)	14 (0.9)	14 (0.9)	20 (1.3)	20 (1.3)	
Unknown or not reported	54 (0.9)	12 (0.8)	14 (0.9)	12 (0.8)	16 (1.1)	
Ethnicity, *n* (%)						
Not Hispanic or Latina	5890 (97.4)	1482 (98.1)	1473 (97.2)	1462 (96.6)	1473 (97.5)	0.15
Hispanic or Latina	160 (2.6)	29 (1.9)	42 (2.8)	51 (3.4)	38 (2.5)	
Unknown or not reported	19 (0.3)	7 (0.5)	2 (0.1)	4 (0.3)	6 (0.4)	
**Health behaviors/status**						
Smoking status, *n* (%)						
Never smoked	3187 (53.3)	1094 (72.8)	970 (64.7)	808 (54.3)	315 (21.2)	< 0.001
Past smoker	2379 (39.8)	405 (27)	513 (34.2)	642 (43.1)	819 (55.1)	
Current smoker	412 (6.9)	3 (0.2)	17 (1.1)	39 (2.6)	353 (23.7)	
Body mass index; kg/m^2^, mean (SD)	28.6 (5.7)	27.1 (5.2)	28.5 (5.4)	29.3 (5.8)	29.3 (6.0)	< 0.001
Total energy expenditure from recreational physical activity; MET‐hours/week, mean (SD)	11.4 (13.2)	13.4 (14.0)	12.0 (14.1)	10.4 (12.2)	9.6, (12.2)	< 0.001
*APOE ε4* carriage	1306 (25.3)	348 (25.7)	307 (23.3)	322 (25.2)	329 (27.1)	0.18

*Note:* Intervals are expressed as (*a*, *b*], where *a* < *x* ≤ *b*; the Q1 interval includes the minimum value.

Abbreviations: EEAA, extrinsic epigenetic age acceleration; IEAA, intrinsic epigenetic age acceleration; MCI, mild cognitive impairment.

### 3.1 Associations of Epigenetic Clocks With Cognitive Outcomes

Over a median follow‐up of 9.3 years (25th and 75th percentiles = 6.1 and 16.1 years, respectively), there were 1307 incident combined MCI/probable dementia events. There was a total of 799 incident MCI and 786 incident probable dementia events when examining each outcome separately (which included 278 participants who developed incident MCI and subsequently probable dementia and were thus included in both analyses). Pearson correlations between epigenetic clocks ranged from 0.03 to 0.96 (Figure S1). Kaplan–Meier curves for the epigenetic clocks and the cognitive outcomes are shown in Figures S2–S6.

**FIGURE 1:**
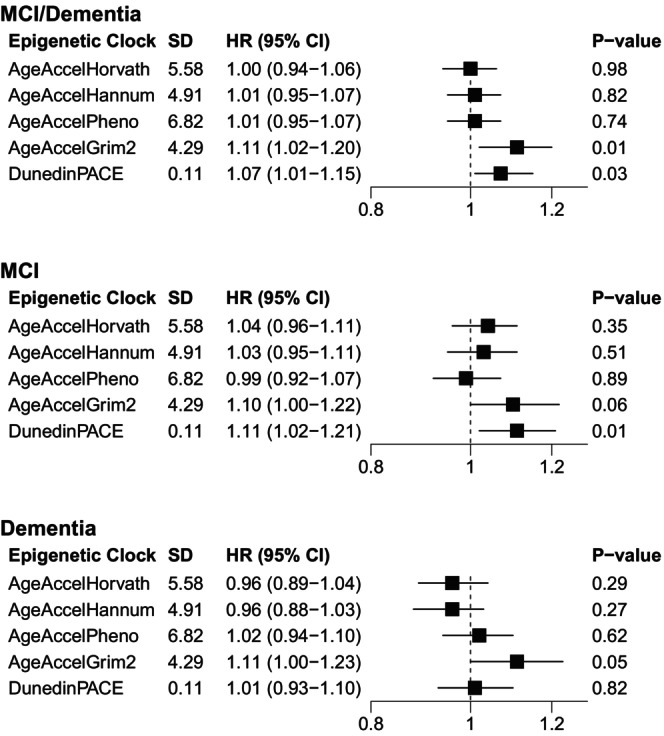
Associations of one‐standard deviation increases in epigenetic clocks with incident mild cognitive impairment (MCI)/probable dementia, MCI, and probable dementia in the Women's Health Initiative Memory Study (WHIMS; *n* = 6069) 1995–2021. EEAA, extrinsic epigenetic age acceleration; IEAA, intrinsic epigenetic age acceleration; MCI, mild cognitive impairment. Intervals are expressed as (*a*, *b*], where *a* < *x* ≤ *b*; the Q1 interval includes the minimum value. There were 1307 incident MCI/probable dementia events, 799 incident MCI events, and 786 incident probable dementia events in an analytic sample of 6069 for analyses with AgeAccelHorvath, AgeAccelHannum, IEAA, EEAA, IEAA.Hannum, AgeAccelPheno, and AgeAccelGrim2. There were 1301 incident MCI/probable dementia events, 797 incident MCI events, and 782 incident probable dementia events in an analytic sample of 6026 for analyses with DunedinPACE.

In fully adjusted models, every one SD increment in DunedinPACE (corresponding to an 11% faster pace of aging compared with the average) was associated with higher risk of the combined endpoint of incident MCI/probable dementia (HR = 1.07, 95% CI = 1.01–1.15; *p* = 0.03) and MCI (HR = 1.11, 95% CI = 1.02–1.21; *p* = 0.01) but not probable dementia (HR = 1.01, 95% CI = 0.93–1.10; *p* = 0.82). Every one SD increment in AgeAccelGrim2 (4.29 years) was associated with higher risk of incident MCI/probable dementia (HR = 1.11, 95% CI = 1.02–1.20; *p* = 0.01) but associations with dementia (HR = 1.11, 95% CI = 1.00–1.23; *p* = 0.05) and MCI (HR = 1.10, 95% CI = 1.00–1.22; *p* = 0.06; Figure 1) separately did not meet criteria for significance. Each one‐standard deviation increment in AgeAccelPheno was not associated with MCI/probable dementia (HR = 1.01, 95% CI = 0.95–1.07; *p* = 0.74), MCI (HR = 0.99, 95% CI = 0.92–1.07; *p* = 0.89), or probable dementia (HR = 1.02, 95% CI = 0.94–1.10; *p* = 0.62; Figure 1). Only the association between AgeAccelGrim2 and the primary outcome of incident MCI/probable dementia remained significant after applying the Bonferroni‐corrected threshold of 0.0167; the association for DunedinPACE was not significant at this threshold. Results from minimally and progressively adjusted models are shown in Table S4.

In secondary analyses, every one SD increment in the PC‐based version of AgeAccelGrim, PCGrimAgeResid (3.34 years), was associated with higher risk of MCI/probable dementia (HR = 1.11, 95% CI = 1.02–1.21; *p* = 0.01) and MCI (HR = 1.13, 95% CI = 1.02–1.26; *p* = 0.02) but not probable dementia in the fully adjusted models (Table S4, Figure S7). No other epigenetic clocks were associated with the cognitive outcomes in the fully adjusted models (Table S4, Figure S7).

In analyses of AgeAccelGrim2 components, higher DNAm PAI‐1 was associated with higher risk of incident MCI/probable dementia (HR = 1.10, 95% CI = 1.04–1.17; *p* = 0.002) and MCI (HR = 1.10, 95% CI = 1.02–1.19; *p* = 0.01) but not probable dementia (Figure S7). Higher DNAm log‐HbA1C was associated with higher risk of MCI/probable dementia (HR = 1.07, 95% CI = 1.00–1.13; *p* = 0.04) but not with MCI or probable dementia separately (Figure S7). Higher DNAm PACKYRS was associated with higher risk of probable dementia (HR = 1.13, 95% CI = 1.01–1.26, *p* = 0.03) but not MCI or MCI/probable dementia. The other AgeAccelGrim2 components were not associated with these outcomes (Figure S7, Table S4). Results from sensitivity analysis that excluded data from 79 women who developed MCI/probable dementia during the first 2 years of follow‐up were consistent in direction and magnitude with results from the main analyses (Figure S8).

**FIGURE 2:**
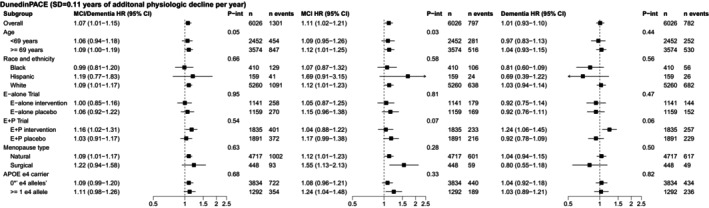
Associations of DunedinPACE with incident mild cognitive impairment (MCI)/probable dementia, MCI, and probable dementia stratified by age, race/ethnicity, hormone therapy regimen, menopause type, and *APOE ε4* carriage in the Women's Health Initiative Memory Study (*n* = 6026), 1995–2021. CI, confidence interval; HR, hazard ratio; MCI, mild cognitive impairment; SD, standard deviation. There were 1301 incident MCI/probable dementia events, 797 incident MCI events, and 782 incident probable dementia events in an analytic sample of 6026 for analyses with DunedinPACE. The sum of separate incident MCI and probable dementia events is higher than the 1301 incident MCI/probable dementia events because 278 participants with incident MCI later had incident probable dementia and are included in both groups. Models adjusted for age, education, smoking, race, ethnicity, hormone therapy trial arm, total energy expenditure from recreational physical activity, body mass index, and white blood cell counts (CD8T, CD4T, natural killer cells, B cells, monocytes, and neutrophils). Models with IEAA, EEAA, and IEAA.Hannum did not include white blood cell counts. Missing covariate data were imputed using multiple imputation by chained equations with the R *mice* package.

**FIGURE 3:**
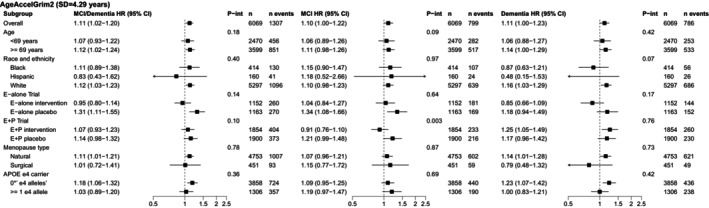
Associations of AgeAccelGrim2 with incident mild cognitive impairment (MCI)/probable dementia, MCI, and probable dementia stratified by age, race/ethnicity, hormone therapy regimen, menopause type, and *APOE ε4* carriage in the Women's Health Initiative Memory Study (*n* = 6069), 1995–2021. BMI, body mass index; CI, confidence interval; E + P, conjugated equine estrogens plus medroxyprogesterone acetate; E, conjugated equine estrogens alone; HR, hazard ratio; *P*‐int, *P*‐interaction. Models adjusted for age, race/ethnicity, education, hormone therapy trial arm, total energy expenditure from recreational physical activity, BMI, and white blood cell counts (CD8T, CD4T, natural killer cells, B cells, monocytes, and neutrophils). Models across strata of race and ethnicity, hormone therapy trial arm, menopause type, and *APOE ε4* carriage do not contain the stratification variable. There were 1307 incident MCI/probable dementia events, 799 incident MCI events, and 786 incident probable dementia events in an analytic sample of 6069 for analyses with DunedinPACE. The sum of separate incident MCI and probable dementia events is higher than the 1307 incident MCI/probable dementia events because 278 participants with incident MCI later had incident probable dementia and are included in both groups.

In stratified analyses, associations of DunedinPACE and AgeAccelGrim2 with MCI/probable dementia generally did not vary by age, race, menopause type, hormone therapy regimen, or *APOE ε4* carriage (Figures 2 and 3, and Table S5).

## 4 Discussion

In a large cohort of older women, we performed the largest prospective study of epigenetic clocks and incident MCI and probable dementia to date. We found that accelerated biological aging as measured by second‐ and third‐generation clocks developed to predict mortality (AgeAccelGrim2) or the pace of aging (DunedinPACE), respectively, was associated with higher risk of incident MCI/probable dementia up to 25 years later independent of chronological age, sociodemographic characteristics, lifestyle behaviors, and health factors. After adjustment for multiple comparisons, only AgeAccelGrim2 remained significantly associated with incident MCI/probable dementia. We performed subgroup analyses by age, race/ethnicity, and *APOE ε4* carriage, as well as menopause type and randomization to hormone therapy, which has not been previously evaluated, and found that associations mostly did not differ across cohort subgroups. Other epigenetic clocks were not associated with incident MCI/probable dementia.

The varying associations of epigenetic clocks with cognitive outcomes observed in the present study could be attributable to the divergent methods used to develop each clock and the corresponding features of aging they may capture. This is apparent in Figure S1 where first‐generation clocks and their corresponding PC‐based or residualized versions showed low to moderate correlations with AgeAccelGrim2 and DunedinPACE, suggesting that these clocks capture different aspects of aging than AgeAccelGrim2 and DunedinPACE. In contrast, AgeAccelGrim2 and DunedinPACE were moderately correlated with each other (*r* = 0.63) suggesting that they capture some shared aspects of biological aging which are more predictive of MCI/dementia than predictors of chronological age. Additionally, epigenetic clocks were developed based on peripheral blood DNAm and may not fully capture aging processes specific to the central nervous system, which may partly explain their modest associations with MCI/probable dementia in our study.

The few large studies of epigenetic clock associations with incident MCI and dementia have also reported associations with DunedinPACE and AgeAccelGrim2, albeit with some discrepancies. In the WHIMS cohort, we previously found that higher DunedinPACE was associated with faster cognitive decline as measured by the TICS‐m test (Nguyen et al. 2024). In the Framingham Heart Study, a 0.12‐unit increment in DunedinPACE was associated with 27% higher dementia risk (HR = 1.27, 95% CI = 1.07–1.49; *n* = 2264; mean age = 65; 54% women; up to 14 years of follow‐up; 151 incident adjudicated dementia events), consistent in direction with our study's findings (Sugden et al. 2022). DunedinPACE was also associated with faster cognitive decline and worse brain structural integrity in the Framingham Heart Study (Savin et al. 2024; Sugden et al. 2022; Whitman et al. 2024). In the ASPirin in Reducing Events in the Elderly study (ASPREE), AgeAccelGrim2, but not DunedinPACE, was associated with higher incident dementia risk among older men (HR = 2.00, 95% CI = 1.15–3.48; *n* = 275; mean age = 74 years; mean follow‐up of 7 years; 37 incident adjudicated dementia events) but not women (HR = 0.96, 95% CI = 0.60–1.53; *n* = 284; mean age = 74 years; 50 dementia events, mean follow‐up of 7 years) (Phyo et al. 2024). DunedinPACE was not associated with dementia risk in either men or women in ASPREE (Phyo et al. 2024). Potential reasons for differences in findings across these studies and ours could be attributable to the larger study population of older women, longer follow‐up (up to 25 years), and larger number of incident MCI/probable dementia events in WHIMS than in prior studies. These prior studies also did not examine MCI/dementia as a combined outcome.

The methods for developing DunedinPACE and AgeAccelGrim2 may also contribute to differences in associations with cognitive outcomes across and within studies. DunedinPACE was developed as a systemic aging indicator from longitudinal data using 19 biomarkers indicative of organ system integrity among 1037 participants between the ages of 26 and 45 years, including markers of cardiovascular, metabolic, renal, pulmonary, immune, and dental system integrity, but not markers of brain/central nervous system integrity (Belsky et al. 2022). DunedinPACE was shown to correlate with cognitive change from adolescence (13 years) to mid‐life (45 years) (Belsky et al. 2022), an age range that encompasses a period of cognitive development as well as age‐related decline. In contrast, cognitive aging among older cohorts such as that examined here (mean age 70 at baseline) reflects different neurophysiological processes related to cognitive function, including those related to neurodegenerative pathologies. As a measure of global physiological aging, DunedinPACE has been shown to more strongly associate with morbidity and mortality outcomes than with dementia later in life (Mavrommatis et al. 2025). The differences in age and phenotype between the sample in which DunedinPACE was developed and to which it has been subsequently applied could contribute to inconsistent associations with cognitive outcomes across studies (Belsky et al. 2022; Conole et al. 2025). While DunedinPACE has been associated with multiple brain endophenotypes in prior studies, these associations may be explained by shared vascular/metabolic pathways and likely do not capture all pathways that contribute to age‐related cognitive change and dementia risk (Whitman et al. 2024).

AgeAccelGrim2 was developed as a mortality estimator, incorporating DNA methylation associated with smoking, chronological age, and plasma proteins associated with mortality (Lu et al. 2022). Unlike DunedinPACE, AgeAccelGrim2 was developed in cohorts that included middle aged and older adults, including the period in life where pathological processes contributing to MCI/dementia are likely accumulating (Alzheimer's Association 2025, n.d.; Lu et al. 2022). Because dementia is associated with earlier mortality, some epigenetic changes associated with dementia may be captured by this clock, even though it was not explicitly trained on dementia outcomes (Liang et al. 2021). Thus, AgeAccelGrim2 may better capture aging‐related biological processes related to dementia than DunedinPACE.

We previously found that both AgeAccelGrim2 and DunedinPACE scores were associated with 15‐year increases in plasma biomarkers of AD, neurodegeneration, and inflammation, in a subset of WHIMS participants (*n* = 873) (Zhang et al. 2025). Higher AgeAccelGrim2 was associated with greater increases in plasma phosphorylated tau at threonine (pTau)‐181 and glial fibrillary acidic protein (GFAP) over time, while higher DunedinPACE was associated with greater increases in pTau‐181, pTau‐217, neurofilament light chain (NfL), and GFAP (Zhang et al. 2025). These findings suggest that the systemic aging processes captured by AgeAccelGrim2 and DunedinPACE may contribute to the development of ADRD‐related neuropathological processes.

While plasma biomarkers of ADRD change continuously over time, or plateau in the case of Aβ 42:40 ratio, with disease progression, MCI and dementia are categorical clinical endpoints. Importantly, while higher levels of these biomarkers are associated with increased risk of dementia, not all people with elevated plasma biomarker levels will develop MCI/dementia (Bilgel et al. 2023; Cogswell et al. 2025). Additionally, MCI and dementia have multifactorial etiology, and not all dementia‐related pathologies are reflected in these few biomarkers. Nevertheless, further investigation of the associations of epigenetic clocks with plasma biomarkers and AD dementia are warranted.

Fewer studies have examined associations of AgeAccelGrim2 or its components with MCI or dementia, as this clock was more recently developed (Lu et al. 2022). Among AgeAccelGrim2 components, DNAm PAI‐1 and DNAm log HbA1C were associated with MCI/probable dementia, while DNAm PACKYRS was associated with probable dementia. PAI‐1 is involved in several aging processes including cellular senescence and inflammation and was found to be higher in postmortem brains with AD than in control brains in a prior study (Barker et al. 2012; Jiang et al. 2023; Khoddam et al. 2025). Diabetes is a dementia risk factor; a meta‐analysis of 144 studies showed that diabetes and higher HbA1c were associated with higher risk of cognitive impairment and dementia (Xue et al. 2019). Smoking is an established risk factor for dementia (Livingston et al. 2024). Taken together, these results suggest that AgeAccelGrim2 captures metabolic and inflammatory processes that contribute to the development of MCI and dementia.

Study strengths include the large population of older Black, Hispanic or Latina, and White women, annual cognitive testing, and rigorously adjudicated MCI and probable dementia. The long follow‐up period and corresponding large number of MCI and probable dementia events in WHIMS enhanced statistical power. We examined first‐, second‐, and third‐generation epigenetic clocks. The WHI collected extensive sociodemographic, behavioral, and health information, enabling comprehensive adjustment for potential confounding.

### 4.1 Limitations

Our study has several limitations. The present study was carried out in a public health randomized trial of women, which could limit the generalizability of results. However, the WHI Hormone Therapy Trials' exclusion criteria were designed to be minimal in order for the study results to be maximally generalizable to women aged 50–79 (Hays et al. 2003). In WHIMS, the study outcome of MCI/probable dementia was categorical and cognitive assessments changed from the in‐person 3MSE to the telephone‐administered TICS‐m after 2007, which could introduce technical noise. However, we previously showed that telephone‐based cognitive assessments were valid and reliable and that telephone‐administered and face‐to‐face cognitive tests yielded equivalent scores (Rapp et al. 2012). Cognitive assessments in WHIMS occurred annually, resulting in interval censoring as the exact timing of MCI and probable dementia events is unknown, a limitation shared by all dementia studies. However, as few participants missed cognitive assessments, we set the date of cognitive assessment as the event date as an approximation, consistent with prior studies (Dunk et al. 2024; Liu et al. 2022). Missing outcome information and informative censoring could occur if participants did not attend cognitive assessments. To reduce this possibility, the WHIMS SCAP was implemented to reduce undercounting of probable dementia cases (Gaussoin et al. 2012, 2019). AD and vascular dementia were not classified in WHIMS after 2007, precluding examination of dementia subtypes. However, AD accounts for approximately 60%–80% of dementia cases, and most individuals with AD have mixed pathology (Alzheimer's Association 2025, n.d.). Although the WHIMS women who were excluded in analyses were largely similar to those in the analytic sample, there were slight differences in age, education, hormone therapy trial regimen, and ethnicity; however, findings were independent of these factors. Future studies inclusive of men and individuals from underrepresented groups are needed to evaluate reproducibility of study findings. Given the observational design, we cannot establish causality. Because we examined multiple epigenetic clocks and not all associations survived correction for multiple comparisons, results should be interpreted with caution.

## 5 Conclusions

In this cohort study of older women, we found that accelerated biological aging as measured by DunedinPACE and AgeAccelGrim2 was associated with higher risk of incident MCI/probable dementia, with AgeAccelGrim2 showing the most robust association. The modest magnitude of observed associations suggests the possibility that stronger epigenetic biomarkers of cognitive aging could be discovered by training clocks specifically on cognitive outcomes, such as MCI and dementia. Overall, these findings provide evidence linking biological aging processes with MCI and dementia in older women.

## Author Contributions

S.N. contributed to the conceptualization and design of the study, conducted data analysis and data interpretation, and led manuscript preparation and manuscript revisions. A.T.L. and S.H. contributed to data analysis and manuscript preparation and manuscript review. M.A.E. contributed expertise for data analysis, data interpretation, and contributed to manuscript review and manuscript revision. S.R.R. provided expertise in design of the study, contributed to data interpretation, and contributed expertise for manuscript preparation. A.X.M. and C.M.N. contributed to data processing, data analysis, manuscript preparation, and manuscript review. A.Z.L. contributed to the conceptualization and design of the study, data analysis, data interpretation, manuscript writing, and manuscript revisions. L.K.M. contributed to the conceptualization and design of the study, data interpretation, and provided expertise for manuscript preparation and revisions. S.M.R. contributed to data analysis, data interpretation, and manuscript writing. K.B. contributed to data generation and provided feedback for manuscript preparation. A.H.S. secured funding, led the conceptualization and design of the study, provided feedback and guidance on data analysis and data interpretation, and contributed and provided guidance for manuscript preparation and manuscript revisions.

## Funding

This study was funded by grant R01AG074345 from the National Institute on Aging, National Institutes of Health. This study was also supported by funds from a program made possible by residual class settlement funds in the matter of April Krueger v. Wyeth Inc., Case No. 03‐cv‐2496 (US District Court, SD of Calif.). Steve Nguyen was also supported by the National Institute on Aging (P01 AG052352, 1K99AG082863‐02). The WHI Program is funded by the National Heart, Lung, and Blood Institute, National Institutes of Health, and US Department of Health and Human Services (R01 HL105065, 75N92021D00001, 75N92021D00002, 75N92021D00003, 75N92021D00004, and 75N92021D00005). The National Heart, Lung, and Blood Institute has representation on the Women's Health Initiative Steering Committee, which governed the design and conduct of the study, the interpretation of the data, and preparation and approval of manuscripts.

## Ethics Statement

This study was approved by the Institutional Review Board of University of California, San Diego.

## Consent

All women provided informed written consent.

## Conflicts of Interest

Mark A. Espeland received support from the Alzheimer's Association (19‐611541). The Regents of the University of California are the sole owners of patents and patent applications directed at epigenetic biomarkers for which Ake T. Lu and Steve Horvath are named inventors. Steve Horvath is a founder and paid consultant of the non‐profit Epigenetic Clock Development Foundation that licenses these patents. Steve Horvath is a Principal Investigator at Altos Labs, Cambridge Institute of Science, a biomedical company that works on rejuvenation. The other authors declare no conflicts of interest.

## Supporting information


**Figure S1:** Pearson correlations between baseline epigenetic clocks in the Women's Health Initiative Memory Study (*n* = 6026).
**Figure S2:** Kaplan–Meier curves for mild cognitive impairment (MCI)/probable dementia, MCI, and probable dementia across tertiles of AgeAccelHorvath in the Women's Health Initiative Memory Study (WHIMS; *n* = 6069) 1995–2021.
**Figure S3:** Kaplan–Meier curves for mild cognitive impairment (MCI)/probable dementia, MCI, and probable dementia across tertiles of AgeAccelHannum in the Women's Health Initiative Memory Study (WHIMS; *n* = 6069) 1995–2021.
**Figure S4:** Kaplan–Meier curves for mild cognitive impairment (MCI)/probable dementia, MCI, and probable dementia across tertiles of AgeAccelPheno in the Women's Health Initiative Memory Study (WHIMS; *n* = 6069) 1995–2021.
**Figure S5:** Kaplan–Meier curves for mild cognitive impairment (MCI)/probable dementia, MCI, and probable dementia across tertiles of AgeAccelGrim2 in the Women's Health Initiative Memory Study (WHIMS; *n* = 6069) 1995–2021.
**Figure S6:** Kaplan–Meier curves for mild cognitive impairment (MCI)/probable dementia, MCI, and probable dementia across tertiles of DunedinPACE in the Women's Health Initiative Memory Study (WHIMS; *n* = 6069) 1995–2021.
**Figure S7:** Associations of epigenetic clocks and AgeAccelGrim2 components with incident mild cognitive impairment (MCI)/probable dementia, MCI, and probable dementia in the Women's Health Initiative Memory Study (WHIMS; *n* = 6069) 1995–2021.
**Figure S8:** Associations of epigenetic clocks and AgeAccelGrim2 components with incident mild cognitive impairment (MCI)/probable dementia, MCI, and probable dementia in the Women's Health Initiative Memory Study after excluding data from 79 women who had incident MCI/probable dementia during the first 2 years of follow‐up (WHIMS; *n* = 5990) 1995–2021.
**Figure S9:** Discriminatory accuracy of epigenetic clocks for incident mild cognitive impairment/probable dementia, (a) alone and (b) with the addition of age, race, and ethnicity.
**Table S1:** Baseline sociodemographic, behavior, and health characteristics by quartiles of DunedinPACE in the Women's Health Initiative Memory Study (*n* = 6026).
**Table S2:** Baseline sociodemographic, behavior, and health characteristics by quartiles of AgeAccelPheno in the Women's Health Initiative Memory Study (*n* = 6069).
**Table S3:** Baseline sociodemographic, behavioral, and health characteristics of 6069 Women's Health Initiative Memory Study women included in the analytic sample versus those excluded (*n* = 1410).
**Table S4:** Associations of epigenetic clocks and AgeAccelGrim2 components with incident mild cognitive impairment (MCI)/probable dementia, MCI, and probable dementia in the Women's Health Initiative Memory Study (WHIMS; *n* = 6069) 1995–2021.
**Table S5:** Associations of epigenetic clocks with incident mild cognitive impairment (MCI)/probable dementia, MCI, and probable dementia stratified by age, race/ethnicity, hormone therapy regimen, menopause type, and *APOE ε4* carriage in the Women's Health Initiative Memory Study (*n* = 6069) 1995–2021.

## Data Availability

The data supporting the present study's findings are available upon reasonable request and review of proposals submitted through the Women's Health Initiative (WHI) study website at https://www.whi.org/propose‐a‐paper. Access is granted to investigators from recognized research institutions.
